# Anti-carcinogenic effects of the phenolic-rich extract from abnormal Savda Munziq in association with its cytotoxicity, apoptosis-inducing properties and telomerase activity in human cervical cancer cells (SiHa)

**DOI:** 10.1186/s12906-015-0530-x

**Published:** 2015-02-12

**Authors:** Guzalnur Abliz, Fatima Mijit, Li Hua, Guzalnur Abdixkur, Tangnur Ablimit, Nurmuhammat Amat, Halmurat Upur

**Affiliations:** Fifth Department of Gynecology, Affiliated Tumor Hospital, Xinjiang Medical University, Urumqi, Xinjiang 830011 China; Traditional Uighur Medicine Institute, Xinjiang Medical University, 393 Medical University Road, Urumqi, Xinjiang 830011 PR China

**Keywords:** Phenolic rich extracts of ASMq, Human cervical cancer, SiHa cells, Apoptosis, Telomerase

## Abstract

**Background:**

Abnormal Savda Munziq (ASMq) is a herbal preparation used in Traditional Uighur Medicine for the treatment cancer. The polyphenol is main compounds contained in ASMq preparation responsible for anticancer effect of ASMq.

**Methods:**

In this study,Real-time quantitative Polymerase Chain Reaction (RT-PCR) assay, MTT assay and flow cytometry were used to investigate the effect of polyphenol of ASMq on cell viability and the potential of the phenolic rich extracts of ASMq to induce apoptosis in human cervical cancer cells SiHa and its effects on telomerase activity were investigated. Cellular morphological change was observed by phase contrast microscopy. The MTT cell viability data revealed that treatment with phenolic rich extracts at 75 ~ 175 μg/ml significantly inhibited the viability and proliferation of cells, and these effects occurred in a concentration-dependent manner and time dependent manner (P < 0.01).

**Results:**

The phenolic rich extracts can induce apoptosis of SiHa cells, can increase the apoptosis rate in a concentration-dependent manner and time dependent manner (P < 0.01). Growth inhibition and apoptosis induction by phenolic rich extracts treatment on SiHa cells was associated with down-regulation of anti-apoptotic Bcl-2 expression and telomerase (P < 0.05) and Survivin expression. In addition, phenolic rich extracts exerted a dose-dependent induction of FHIT expression.

**Conclusion:**

These results suggest that phenolic rich extracts may have anti-tumor effects in human cervical cancer through cytotoxicity, apoptosis-inducing properties and telomerase activity.

## Background

Cervical cancer is one of the most common cancers among women worldwide, second only to breast cancer, and disproportionately affects women in developing countries [1,2]. Although the worldwide incidence has decreased, primarily because of the widespread use of cervical screening programs, it is still the second most common gynecological malignancy. Furthermore, the number of cervical cancer mortality cases in China is among the highest in the world [3].

Cancer is a multifactorial and multistep disease caused by the accumulation of multiple hits which involves genetic and epigenetic alterations leading to aberrant expression of genes involved in initiation, progression and promotion of carcinogenesis [4]. It is now well accepted that apoptosis is a physiological phenomenon that plays an important role in the regulation of tissue development and maintenance of homeostasis, and elimination of damaged cells. Apoptosis is tightly regulated by a number of gene products that promote or block cell death at different stages. Deregulation of apoptosis has been shown to contribute to the pathogenesis of a number of human diseases including cancer [5,6]. There are apparently many factors that have been demonstrated to be critical in the regulation of apoptosis, including Bcl-2 family, FHIT and Survivin, involved in the apoptotic process through the expression of genes, and the characterization of the function of these gene products will help to define the process of cell death at the molecular levels [7-9].

Telomeres are essential units that prevent the loss of genetic information. Telomere dysfunction in turn induces a permanent proliferation arrest known as senescence and cell death [10]. Telomerase plays a critical role in cell immortality and tumor formation, it has been suggested that telomerase reactivation is a rate-limiting step in cellular immortality and carcinogenesis, and that telomerase repression can act as a tumor-suppressive mechanism [11]. Studies have indicated that activation of telomerase enzyme and telomere stabilization is an important step in tumorigenesis. Inhibition of telomerase activity results in the shortening of telomeres and finally leads to the crisis state resulting in apoptosis [12]. A connection between telomerase activity and resistance to apoptosis has also been established [13].

More than 80% of the world’s population makes use of complementary and alternative medicines which include herbalism and botanical medicines [14]. Traditional Medicine appears to play an important role in human health and in the development of certain diseases, especially cancer. Strong and consistent epidemiological evidences suggest that a diet enriched with naturally occurring substances significantly reduces the risk for many cancers. Many drugs used for the treatment of cancer have been discovered from medicinal plants [15]. A major group of these products include pigments, vitamins, phenolic lactones, flavonoids, tannins and alkaloids [16,17]. One group of promising phytochemicals is polyphenols that inhibit carcinogenesis in several models [18]. The effects of dietary phenolic compound are of great interest currently due to their antioxidative and possible anticarcinogenic activities. Flavonoids and other phenolic compounds derived from fruits and berries have been shown to induce apoptotic pathways and to suppress proliferation of various types of cancer cells [19-22].

Abnormal Savda Munziq (ASMq), one of the Uighur medicinal herbal preparations, is widely distributed in the Xinjiang region of China. It has long been used in Traditional Uighur Medicine for the treatment of several diseases such as cancer, diabetes, cardiovascular diseases or chronic asthma [23]. Recent studies have shown that phenolic compound of Abnormal Savda Munziq (ASMqp) can significantly inhibit the growth and viability of Hep G2 human hepatoma cell line [24]. ASMqp has also been reported to scavenge free radicals [25]. In addition, some species included in ASMq were reported to show in vitro inhibitory activity on different tumor cell lines [26-31]. A phenolic fraction with high anti-proliferative activity on HL-60 cells was obtained from ASMq through the bioassay-guided fractionation process. The results of the cell viability assay, quantification assay and the TLC and LC-MS analyses on ASMq fractions revealed that the main bioactive components with anti-proliferative activity against HL-60 cell line could be phenolic compounds [32].

The present work was undertaken to investigate the underlying mechanism involved in the induction of apoptosis by phenolic compounds from ASMqp in human cervical cancer cell SiHa with special emphasis on its role in regulation of apoptosis, including expression of Bcl-2 family, FHIT and Survivin, as well as inhibiting telomerase activity. The primary objective of our study is to establish a possible association of apoptosis with suppression of telomerase activity. The present study was undertaken to investigate whether the combinatorial chemopreventive effect of phenolic-rich extract from ASMq on human cervical cancer cell SiHa is mediated by decreasing cell proliferation, inducing apoptosis, the apoptosis-associated proteins Bcl-2 family, FHIT and Survivin, and inhibiting telomerase activity.

## Methods

### Plant materials

*Pobumuguo* (fruits of *C. dichotoma*), *Niushecao* (whole plant of *A. italica*), *Gancao* (root of *G. uralensis*), *Tiexianjue* (whole plant of *A. capillus-veneris*), *Dijincao* (whole plant of *E. humifusa*), *Hongzao* (fruits of *Z. jujuba*), *Xunyicao* (aerial part of *L. angustifolia*), *Xiaohuixiang* (fruits of *F. vulgare*), *Mifenghua* (whole plant of *M. officinalis*) and *Citang* (sugar *secretion from A. pseudoalhagi*) were purchased from Xinjiang Hospital of Traditional Uighur Medicine, Urumqi, China in September 2007. The plant material used was unprocessed.

### Preparation of abnormal Savda Muziq (ASMq)

ASMq was prepared according to this procedure. The mixture was decocted in boiling water in a ratio of 1:10 (w/v) for 3 h. After filtration, the residue was reextracted for 3 h, two times in the same volume of boiling water. The resulting crude extract was filtered, evaporated to dryness under reduced pressure and pulverized. The obtained powder was used for this study. The yield was 39.9% (w/w) with respect to the total mass of dry materials.

### Extraction of polyphenolic compounds from beans from ASMq

The protocol used to obtain polyphenolic rich extracts from bean samples was based on the previously method [32]. Dry extract of ASMqp (1500 g) was dissolved in 1050 mL hot H_2_O (60°C) and filtered.1.5 volumes of 95% EtOH were added; the mixture was stirred for 20 min, and then allowed to stand for 24 h. The resulting supernatant layer was filtered, concentrated under reduced pressure to 350 mL and subjected to column chromatography (125 cm × 5 cm) on polyamide resin. The column was eluted first with H_2_O dest. (3BV), followed by 60% EtOH. The fraction eluted with H_2_O dest was discarded, while the fraction eluted with 60% EtOH was evaporated under reduced pressure to dryness to obtain phenolic rich extract (15.4 g). The yield (w/w) of polyphenol fraction was 3.49% with respect to the dry weight of ASMq.

### Cell lines and culturing

Human Cervical cancer SiHa cells were obtained from the Shanghai Cell Bank of Chinese Academy of Sciences and maintained in our laboratory. The cells were grown as monolayers in DMEM medium supplemented with 2 m Mglutamine, antibiotics (100 U/ml penicillin A and 100 U/ml streptomycin), and 10% heat-inactivated fetal bovine serum (FBS), and maintained at 37°C in a humidified incubator containing 5% CO_2_ + 95% air. All cells were passed twice weekly and routine examination was also done for mycoplasma contamination. Cells in logarithmic growth phase were used for further experiments.

### Chemicals and reagents

DMEM medium and fetal calf serum (FCS) were purchased from GIBCO compnay (USA), The apoptosis detection kit was from Becton Dickinson and Company (San Jose, CA, USA). 3-(4,5-dimethyl-2-thiazyl)-2, 5-diphenyl-2H-tetrazolium bromide (MTT), tyripsin and DMSO were from Amresco (USA), fix and perm kit was from Caltag laboratories, Beckman. Coulter USA), PE Anti-Bcl-2 Antibody was from Invitrogen Co., Ltd. (Shanghai, China), FITC Anti- Polyclonal antibodies for polymerase was from Bioss Technology Development Co., Ltd., (Beijing China). Other chemicals were commercially available reagent grade or ultrapure grade.

### Method

#### MTT assay

Cytotoxicity was measured by MTT assay. SiHa cells (1 × 10^5^/well) in 100 ul DMEM were plated in 96-well plates and incubated for 24 h to allow the cells to attach, before treatment extract. Extract was dissolved in DMSO and the cells were treated with 75 μg/ml, 100 μg/ml, 125 μg/ml, 150 μg/ml, 175 μg/ml concentration of extract for 24, 48,72 h and 96 h, Cells treated with 0.1% DMSO served as a negative control. After incubation for specified time at 37°C in a humidified incubator, 20 ul MTT (5 mg/ml in PBS) was added to each well and incubated for 4 h, after which the plate was centrifuged at 1800 g for 5 min at 4°C. After careful removal of the medium, 150 ul of buffered DMEM was added to each well, and plates were shaken. The absorbance was recorded on a microplate reader (Bio Rad Laboratories, Hercules, CA, USA) at the wavelength of 490 nm. The effect of each compound on growth inhibition was assessed as percent cell viability where vehicle-treated cells were taken as 100% viable. The experiments were performed in triplicate and results were described as average of A. For morphological study, the cells were treated with WECM for 48 h and directly photographed with an inverted microscope.

#### Assessment of apoptosis by flow cytometric analysis

Cytotoxicity was measured by MTT assay. Cells were counterstained with PI as a vital dye to distinguish between apoptotic (annexin V positive, PI negative) and necrotic (annexin V positive, PI positive) cells. SiHa cells (1 × 10^5^/well) in 2 ml DMEM were plated in 6-well plates and incubated for 24 h to allow the cells to attach, before treatment extract. Extract was dissolved in DMSO and the cells were treated with 75 μg/ml, 100 μg/ml, 125 μg/ml, 150 μg/ml concentration of extract for 48 h, Cells treated with 0.1% DMSO served as a negative control (0 μg/ml) and digested for 4 minutes with 0.25% trypsin, collect the cells was washed with 4°C PBS twice, centrifugation at 1500 r/min for 10-minute, were resuspended in 100 ul of binding buffer and incubated with 5 μl FITC-conjugated Annexin-V and 5 μl PI for 15 minutes at room temperature in the dark, add 400 μl binding buffer each tube, and were then immediately analyzed by flow cytometry (FACScan’BD Immunocytometry Systems, Mountain View, CA). A minimum of 10,000 events was acquired for each sample.

#### Flow cytometric analysis of bcl-2 and telomeres

Cytotoxicity was measured by MTT assay. Cells were counterstained with PI as a vital dye to distinguish between apoptotic (annexin V positive, PI negative) and necrotic (annexin V positive, PI positive) cells, respectively. SiHa cells (1 × 10^5^/well) in 2 ml DMEM were plated in 6-well plates and incubated for 24 h to allow the cells to attach, before treatment extract. Extract was dissolved in DMSO and the cells were treated with 75 μg/ml, 100 μg/ml, 125 μg/ml, 150 μg/ml concentration of extract for 48 h, Cells treated with 0.1% DMSO served as a negative control (0 μg/ml). and digested for 4 minutes with 0.25% trypsin, collect the cells was washed with 4°C PBS twice, centrifugate at 1500 r/min for 10-minute, discard supernatants. Then the cells were resuspended in 1 ml of PBS, then the cells were pipetted into two flow cytometry test tube, one tube served as a negative control, one tube served as a assay tube, SiHa cells is 1 ~ 5 × 10^5^ each tube, and add each tube Fix&Perm reagent 1 100 ul, shaked and then standing for 15 min at room temperature, add 4 ml PBS, centrifugated at 1500 r/min for 5 min, discard supernatants. Then add each tube Fix &Perm reagent 2 100 ul and standing for 15 min at room temperature. Then add Anti-Telomerase (TE)/FITC antibody 2 ul and anti-Bcl/PE antibody to test tube, shaken slightly, standing for 15 min at room temperature in the dark. Then add 4 ml PBS to each tube, centrifugate at 1500 r/min for 5 min, discard supernatants. The cells were resuspended in 1 mlul of PBS, and were then immediately analyzed by flow cytometry (FACScan’BD Immunocytometry Systems, Mountain View, CA). A minimum of 10,000 events was acquired for each sample. Data were analyzed using the Flow Jo software (Treestar, San Carlos, CA).

#### Measurement of FHIT and Survivin expression by real-time PCR

Following treatment for 48 h, cells grown in 60 mm Petri dishes were washed with ice-cold PBS and 1 ml of trizol reagent (Invitrogen Inc, Carlsbad, CA) was added and flushed gently to disrupt the cells, the lysates were collected and mixed with 200 μl of chloroform by inversion. The tubes were then centrifuged at 10,000 rpm for 15 min at 4°C. The aqueous phases from the tubes were collected and the RNA precipitated using 700 μl of isopropanol and centrifuged at 12,000 rpm for 10 min at 4°C. The pellets were washed twice with 75% ethanol, centrifuged at 7500 rpm for 10 min at 4°C and air-dried for about 20–40 min. The pellets were resuspended in 20 μl of DEPC treated water. The purity of RNA was checked by OD260/280 of RNA samples (N1.8). The quality of the RNA was analyzed by agarose gelelectrophoresis. Reverse transcription was then performed on the 1 μg of total RNA using the ExScript RT reagent Kit (TaKaRa).Isolated total RNA (1 μg) was reverse-transcribed to cDNA in a reaction mixture containing 4 μl of 5 × reaction buffer, 1 μl of PrimeScriptTM RT Enzyme Mix, 1 μl of Oligo dT Primer (50 μM), 1 μl of Random 6 mers (100 μM) and Rnase Free dH2O, in a total volume up to 20 μl. The reaction mixture was incubated at 37°C for 45 min and the reaction was terminated by heating at 85°C for 5 sec. The resultant cDNA was diluted 10 times and use for RT-PCR amplification.

All oligonucleotide primers were purchased from Sigma Genosys, India. Details about the primers are given in Table 1. Quantitative real-time RT-PCR for the FHIT and Survivin mRNA was performed on an ABI PRISM127100 Sequence Detection System (Applied Biosystems, Foster City, CA, USA) using SYBR Green Master Mix (Takara Biotechnology Co. Ltd., Dalian, China). For normalization, glyceraldehyde-3-phosphate dehydrogenase (GAPDH) was used. The final reaction volume was 20 μl, containing 10 μl × SYBR12 Premix ExTaqTM, 0.5 μl Forward Primer (10 μM), 0.5 μl Reverse Primer(10 μM) 2.0 μl cDNA and 7 μl dH_2_O. Cycling conditions were as follows: initial denaturation at 95°C for 10 s, followed by 40 cycles of 60°C for 30 s and 59°C for 31 s. After PCR, a dissociation curve analysis was done. Relative gene expression was calculated using the 2^−△△^CT method with pooled cDNA from all samples as a reference.

**Table 1:** **The primers used in this study for semiquantitative RT-PCR assay**

**Gene**	**Primer primer sequence**
β-actin	F: 5′- TGGCACCCAGCACAATGAA-3′
	R: 5′- CTAAGTCATAGTCCGCCTAGAAGCA-3′
FHIT	F: 5′- GCAGCTCTGCGGGTCTACTTTC-3′
	R: 5′- TCTTCAAACTGGTTGGCAATAGCTC-3′
Survivin	F: 5′- GTCCGGTTGCGCTTTCCTT-3′
	R: 5′- CGCAGTTTCCTCAAATTCTTTCTTC-3′

F: Forward primer, R: Reverse primer.

### Statistical analysis

All values were presented as means ± SEM and evaluated for statistical significance with one-way ANOVA followed by Duncan’s multiple range test. P values less than 0.05 were considered significant.

## Results

### Cytotoxicity and inhbiitory activity of ASMq extract on cell morphology and cell proliferation

**Figure 1:**
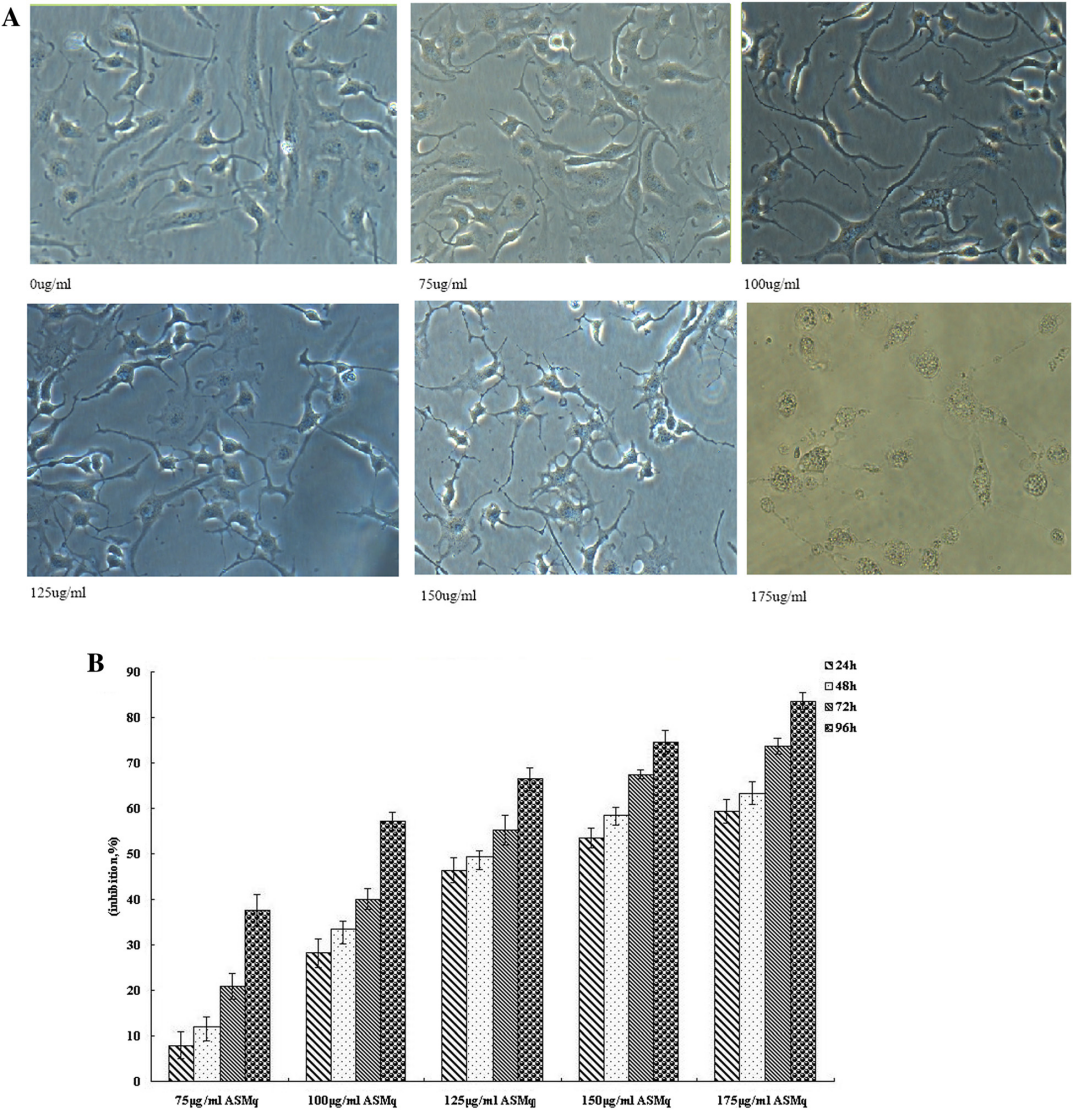
**Morphologic changes of Siha cells treated with phenolic rich extracts of ASMq and inhibition of the cell growth of phenolic rich extracts of ASMq on Siha cells. A**: Cells were incubated with varying concentrations of phenolic rich extracts of ASMq for 48 h, observed and photographed with an inverted microscope. (magnification × 200). **B** Inhibitory effect of ASMp on proliferation of of Siha cells in dose-dependent manner and time dependent manner. Mean absorbance observed in the MTT assay after treatment of 24, 48 72 h and 96 h with 75 ug/ml, 100 ug/ml, 125 ug/ml and 150 ug/ml of ASMqp. Growth inhibition was calculated relative to negative control, and the data represents the mean ± SE of three experiments each conducted in triplicate.

To evaluate the effects of ASMp on cell proliferation, we investigated the effects of ASMqp on cell growth of Siha cells. Cells were exposed to increasing doses of ASMq extract for 24 h, 48 h, 72 h and 96 h cell viability was determined by the MTT assay. As shown in Figure 1, cell viabilities of Siha were markedly decreased after exposure to ASMq extract in a dose-and time-dependent manner. The proliferation of Siha cells was reduced by about 50% after 24 h of exposure to 150 μg/ml and 175 μg/m of ASMq extract. The inhibition ratio is 83.55% after 96 h of exposure to 175 μg/ml of ASMq extract. The IC_50_ is 134.51 ± 2.55 μg/ml after 48 h of exposure to of ASMq extract. In addition, direct observation using an inverted microscope revealed that numerous morphological changes occurred in cells treated with of ASMq extract. In particular, cell shrinkage, condensation of cytoplasm, and formation of cytoplasmic filaments, chromosomal condensation and formation of apoptotic bodies appeared in a concentration-dependent manner after ASMq extract treatment. After exposed to 120 μg/mL of ASMq extract for 24 h, cells began to shrink with diminished cytoplasm and gradually detached from the plate wall. After 72 h, cells had a round shape and karyopyknosis appeared (Figure 1).

### Apoptosis is induced by ASMqp in Siha cells

**Figure 2:**
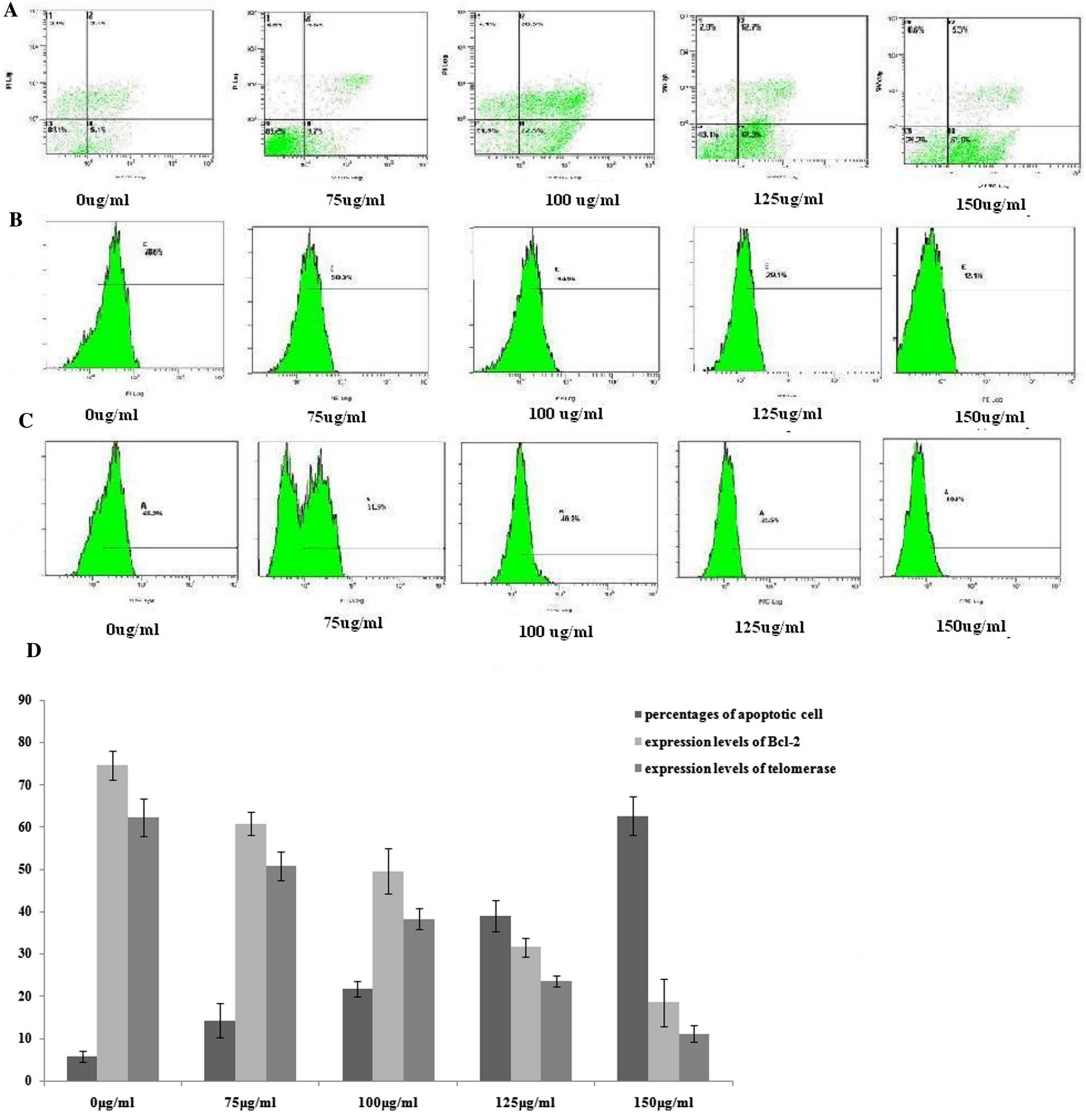
**Apoptosis-inducing, inhibition of Bcl-2 expression and telomerase activity by phenolic rich extracts of ASMq.** Extracts of ASMq treatment in Siha cells during treatment with 75 ug/ml, 100 ug/ml, 125 ug/ml and 150 ug/ml of extracts of ASMq for 48 h. **A**: The cells were sampled and stained with Annexin V-FITC/PI to quantify the degree of apoptosis induced by ASMqp, the cells were evaluated and analyzed by flow cytometry. Values represent mean ± S.D. (n = 10000). **B**, **C**: The cells incubated with Anti-Telomerase (TE)/FITC antibody and anti-Bcl/PE antibody after treatment with ASMqp, and were then analyzed by flow cytometry. **D**: Values represent mean ± S.D. (n =10000).

The results of apoptosis ratio found after 24 h of treatment with 0, 75, 100, 125 and 150 ug/ml of of ASMq extract are given in Figure 2, where we can see that different concentration of of ASMq extract tested can induce apoptosis. There were significant differences observed in the proportion of of ASMq extract Annexin V-positive cells between treated and vehicle-treated cell groups. The three independent measurements made after 48 h of exposure to 150 ug/mL of of ASMq extract revealed that the percentages of apoptotic cells (Figure 2A and D). There were 14.20 ± 4.06%, 21.70 ± 1.84%, 39.13 ± 3.68%, 62.73 ± 4.49% Annexin V-positive cells among the cells treated with 75, 100, 125 and 150 ug/ml of ASMq extract respectively for 48 h, and compared to the vehicle-treated cells (5.39 ± 1.31%), indicating that ASMqp induced apoptosis in the Siha cells in a dose-dependent manner.

### ASMqp down-regulated expression of Bcl-2 and telomerase activity

To explore the possible mechanism by which of ASMq extract induced apoptosis in Siha cells, the expression of both Bcl-2 and telomeres activity was analyzed by flow cytometry. As indicated in Figure 2B, C and D). Significant changes in expression of Bcl-2 and telomeres were noted in Siha cells treated with different concentration of ASMq extract for 48 h, of ASMq extract markedly down-regulated the expression levels of Bcl-2 and telomerase in a concentration-dependent manner.

### ASMqp regulate of expression of FHIT and Survivin

**Figure 3:**
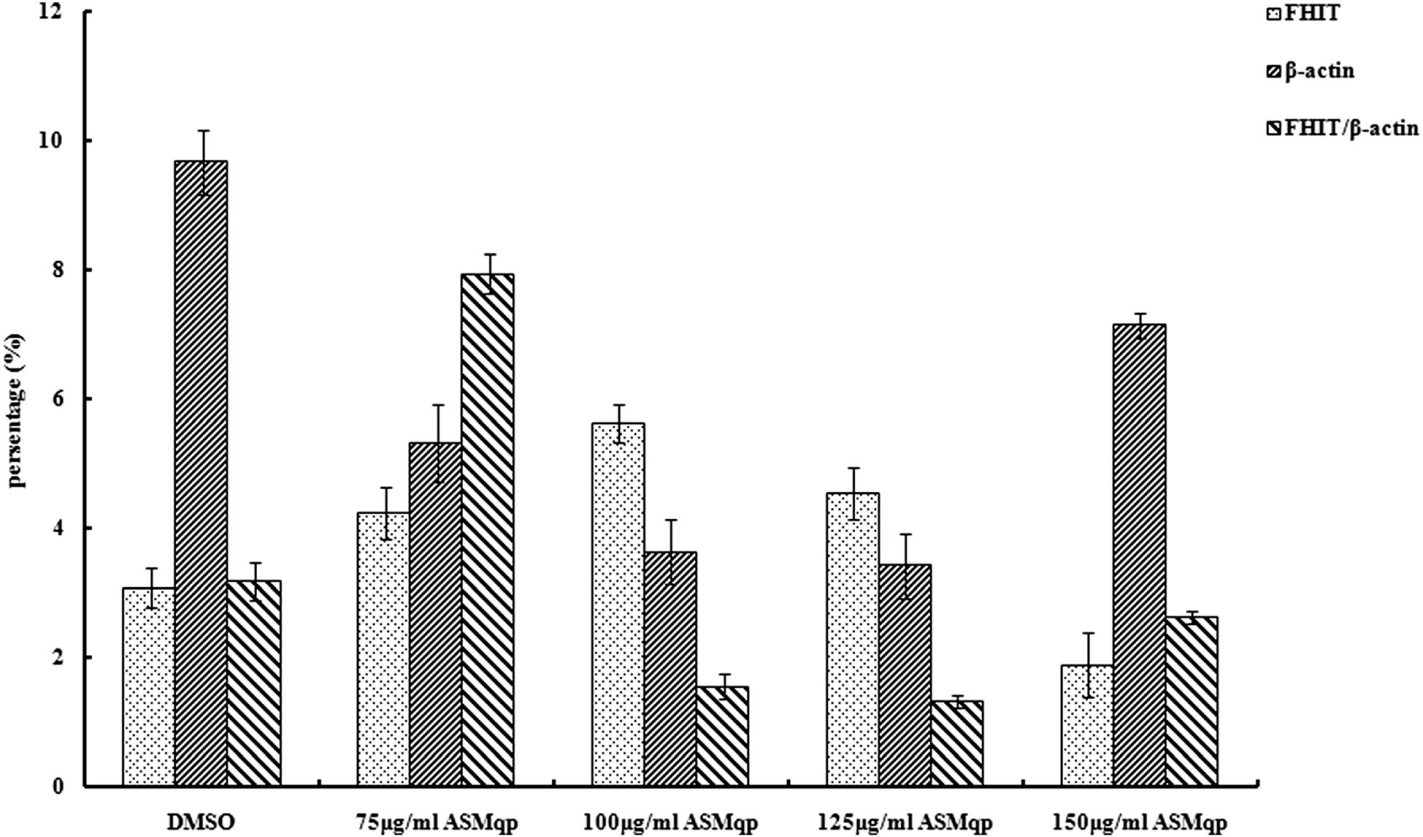
**Effect of ASMqp on expression of FHIT in Siha cells was measured using a RT-PCR analyses, β-Actin was used as an internal loading control.** The experiment was quantified by densitometry and represented as the indicated expression of FHIT/β-actin ratio. Representative results from three independent experiments are shown. Data are expressed as means ± SD of three independent experiments.

**Figure 4:**
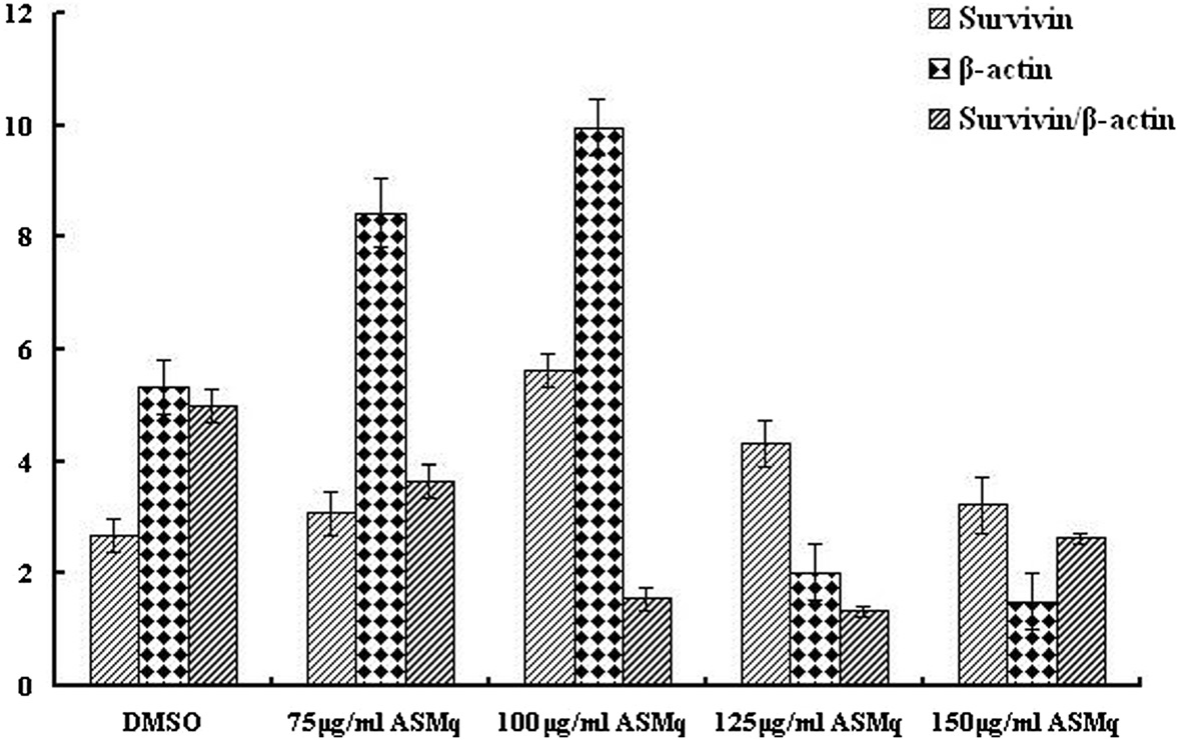
**Effect of ASMqp on expression of Survivin in Siha cells was measured using a RT-PCR analyses, β-Actin was used as an internal loading control.** The experiment was quantified by densitometry and represented as the indicated expression of Survivin/β-actin ratio. Representative results from three independent experiments are shown. Data are expressed as means ± SD of three independent experiments.

To investigate the mechanism responsible for the apoptosis induced by ASMq extract in Siha cells, we analyzed the FHIT and Survivin gene expression by RT-PCR analys is using specific primers (Table 1). As shown in Figures 3 and 4. the gene expression of FHIT up-regulated in Siha cells after treatment by ASMq extract for 48 h. This indicated that there was increase in the FHIT/β-actin ratio. But the gene expression of Survivin down-regulated in dose dependently in Siha cells after treatment by f ASMq extract for 48 h. This indicated that there was decrease in the Survivin/β-actin ratio.

## Discussion

Consistency in both composition and biological activity are critical requirements for safe as well as effective development and use of medicinal herbs. Flavonoids are yphenolic compounds that occur naturally in various plant species. They are utilized mainly as a source of starting material in the pharmaceutical and food industries and show numerous biological activities of interest, for example, antioxidant capacity, anti-inflammatory action and stimulation of the immune system [33,34]. In recent years, identification of effective chemopreventive polyphenols in diets or dietary supplements for human use is of much interest. Apoptosis plays a crucial role in eliminating the mutated hyperproliferating cells from the system. Thus, induction of apoptosis in tumor cells may be considered as a protective mechanism against development and progression of cancer [35].

In this work, we have demonstrated total polyphenolic compounds of traditional Uighur medicine preparation Abnormal Savda Munziq, in a dose-and time-dependent manner inhibited cellular proliferation of Siha cells by MTT assay. Morphological characterization of phenolic rich extracts -treated cells revealed that the mode of action of cell death is mediated through induction of apoptosis. Typical features of apoptosis like cell shrinkage, cytoplasmic condensation, and formation of cytoplasmic filaments, chromosomal condensation and formation of apoptotic bodies of phenolic rich extracts -treated cells clearly evident from inverted microscope study. Flow cytometry analysis showed that apoptotic cells gradually increased in a dose and time dependent manner after the cells were treated with concentrations of phenolic rich extracts of ASMq for 48 h. To clarify the mechanisms of apoptosis caused by phenolic rich extracts of ASMq, we detected expression of the common apoptosis-dependent related Bcl-2, telomerase activity, FHIT expression and survivin expression. Flow cytometry and RT-PCR analysis revealed that bcl-2 expression, telomerase activity and survivin expression were down-regulated remarkably while FHIT expression up-regulated.

Apoptosis plays a crucial role in eliminating the mutated hyperproliferating cells from the system. Recently, inducers of apoptosis have been used in cancer therapy and activation of apoptosis pathways is a key mechanism by which cytotoxic drugs kill tumor cells and now been considered as an important method of assessment for the clinical effectiveness of many anti-tumor drugs [36].Apoptosis is tightly regulated by a number of gene products that promote or block cell death at different stages. One of the major genes that regulates apoptosis is the Bcl-2family. It has been reported that Bcl-2 members protect against multiple signals that lead to cell death, whereas Bax members induce apoptosis indicating that Bcl-2 family regulates a common cell death pathway and functions at a point where various signals converge [37,38]. The Bcl-2 family is the most extensively studied and perhaps the most important target for chemopreventive agents [39,40].

Telomerase is a specialized reverse transcriptase that synthesizes and preserves telomeres, thereby playing a key role in regulating the lifespan of cell proliferation. Telomerase activity is critically involved in cell development, aging and tumorigenesis, and is required for self-renewal and proliferative expansion in a number of cell types, including most cancer cells [41]. Most human cancers have short telomeres and express high levels of telomerase activity as compared to normal tissue [42]. It has been thought that telomerase activation might be a critical step in cellular immortalization. According to the telomere hypothesis for replicative senescence, genetic experiments using an ineffective form of human telomerase have demonstrated that telomerase inhibition can result in telomere shortening followed by growth arrest and apoptosis [43,44], supporting the potential use of telomerase inhibition for cancer therapy. Inhibition of telomerase results in telomere shortening, repressed proliferation and altered cell cycle that result in apoptosis [45]. Telomerase has been shown in several studies to be a potentially sensitive biomarker for early cancer screening [46]. Also, it is believed that the modification of telomerase activity may be a potential therapeutic modality for the treatment of human cancers. It was reported that the over-expression of Bcl-2 in human cervical and colorectal carcinoma cells resulted in an increased telomerase activity and a resistance to apoptosis, indicating a link between Bcl-2 expression and telomerase activity in human cancer cells [47]. As shown in Figure 2, ASM extract induced a concentration-dependent inhibition of telomerase activity with down-regulation of the expression of Bcl-2. The results suggested that repression of telomerase activity by KRG extract was associated with down-regulation of Bcl-2 expression.

FHIT is a tissue-specific tumor suppressor gene, and its inactivity is often associated with the occurrence and development of cancer, especially epithelial cancer, including cervical carcinomas [48]. It has been reported that re-expression of FHIT in a variety of human cell lines results in growth inhibition and apoptosis induction. Previous studies reported that a reduction of FHIT was positively correlated with the rate of distant metastases and worse prognosis. Over-expression of FHIT is directly proportional to the apoptotic rate in the tumors examined [49]. Furthermore, FHIT overexpression produces alteration in cell cycling properties, as well as reduction of the tumorigenic potential in nude mice [50]. FHIT plays a critical role in FHIT-induced apoptosis, occurring through inactivation of the Survivin-Caspase signal pathway in the development of human CRC. Restoration of FHIT expression induced apoptosis in all FHIT-negative cell lines and SiHa cells among cervical carcinomas [51]. Results indicate that paclitaxel-induced apoptosis enhanced by FHIT expression in lung cancer cells might be associated with modulation of Bcl-2-caspase signaling [52].

Therefore, FHIT replacement or therapeutic activation of the FHIT pathway could contribute to cancer prevention. Survivin is a member of a new protein family known as the ‘inhibitors of apoptosis’, blocks apoptosis by interacting with and inactivating the proapoptogenic mitochondrial protein SMAC (second mitochondria-derived activator of caspase) and by inhibiting caspases [53]. It is present during embryonic development but absent from terminally differentiated adult tissues, and is prominently expressed in transformed cell lines and most tumour types [54], including cervical cancer [55].

Recently, survivin has attracted the attention of scientists as a candidate biomarker because of its unique distribution. Some studies have demonstrated that FHIT activation is associated with inactivation of the PI3K-Akt-survivin signal pathway and increased Bcl-2 expression during in Fhit-induced apoptosis. Akt has been implicated as a major factor in many types of cancer. FHIT has been shown to modulate the Akt-survivin pathway by inhibiting the activity of Akt, a key effector in the phosphatidylinositol 3-OH kinase (PI3K) pathway. In this study, apoptotic cell death was accompanied by FHIT up-regulation and Survivin down-regulation, suggesting the ASMqp-induced apoptosis is probably mediated through the Akt-survivin pathway and Bcl-2 related pathways.

## Conclusions

In conclusion, the results of the present study reveal that ASMqp inhibits Siha cell proliferation and induce apoptosis through the proapoptotic and antiapoptotic genes. This provides a molecular basis for understanding the chemopreventive effect of ASMqp that might be ideal candidates to induce effective apoptosis in human cervical cancer cells. However, presently the detailed mechanism underlying the antiproliferative role of ASMqp has been delineated in case of Siha cells only. More work is required to find out the precise role of phenolic rich extracts of ASMq in different types of cervical cancer a cells to understand whether the action of these polyphenols is cell line specific general mechanism. These observations will add new light in the field of developing therapeutic strategies for cancer in the near future in Uighur Medicne. To our knowledge, this is the first report of the induction of apoptosis and its mechanism is induced by phenolic rich extracts of ASMq, an important constitution in the ASMq.
